# Substrate sequence selectivity of APOBEC3A implicates intra-DNA interactions

**DOI:** 10.1038/s41598-018-25881-z

**Published:** 2018-05-14

**Authors:** Tania V. Silvas, Shurong Hou, Wazo Myint, Ellen Nalivaika, Mohan Somasundaran, Brian A. Kelch, Hiroshi Matsuo, Nese Kurt Yilmaz, Celia A. Schiffer

**Affiliations:** 1 Biochemistry and Molecular Pharmacology, UMass Medical School, Worcester, MA 01655 USA; 2 Leidos Biomedical Research, Inc., Frederick National Laboratory for Cancer Research, Frederick, MD 21701 USA

## Abstract

The APOBEC3 (A3) family of human cytidine deaminases is renowned for providing a first line of defense against many exogenous and endogenous retroviruses. However, the ability of these proteins to deaminate deoxycytidines in ssDNA makes A3s a double-edged sword. When overexpressed, A3s can mutate endogenous genomic DNA resulting in a variety of cancers. Although the sequence context for mutating DNA varies among A3s, the mechanism for substrate sequence specificity is not well understood. To characterize substrate specificity of A3A, a systematic approach was used to quantify the affinity for substrate as a function of sequence context, length, secondary structure, and solution pH. We identified the A3A ssDNA binding motif as (T/C)TC(A/G), which correlated with enzymatic activity. We also validated that A3A binds RNA in a sequence specific manner. A3A bound tighter to substrate binding motif within a hairpin loop compared to linear oligonucleotide, suggesting A3A affinity is modulated by substrate structure. Based on these findings and previously published A3A–ssDNA co-crystal structures, we propose a new model with intra-DNA interactions for the molecular mechanism underlying A3A sequence preference. Overall, the sequence and structural preferences identified for A3A leads to a new paradigm for identifying A3A’s involvement in mutation of endogenous or exogenous DNA.

## Introduction

The APOBEC3 (short for “apolipoprotein B mRNA editing enzyme, catalytic polypeptide-like”) family of human cytidine deaminases provides a first line of defense against many exogenous and endogenous retroviruses such as HIV-1 and the retro-element LINE-1^1–6^. APOBEC3 (A3) proteins restrict replication of retroviruses by inducing hypermutations in the viral genome^7^. A3s deaminate deoxycytidines in ssDNA into uridines during reverse transcription. This results in G to A hypermutations, as adenosines are transcribed across from uridines during second strand DNA synthesis. While all A3 enzymes deaminate deoxycytidines in ssDNA, they have differential substrate specificities that are context dependent, resulting in altered frequencies of mutation for the deoxycytidines. Some A3s deaminate the second deoxycytidine in a sequence containing CC while others deaminate deoxycytidine in a TC context^8–10^. However, not every cognate dinucleotide motif (CC or TC) in the ssDNA of the HIV genome is deaminated^11^. Nevertheless, hypermutation in a viral genome results in defective proteins and proviruses, thus decreasing the probability of further viral replication^12^.

Beyond restricting viral replication, the ability of A3s to deaminate deoxycytidines in ssDNA have made A3s a double-edged sword. When overexpressed, A3s can mutate the host genome resulting in a variety of cancers. The identities and patterns of the mutations observed in cancer genomes can define the source of these mutations. Recently, the search for the deaminase(s) responsible for kataegic mutations found in breast cancer was narrowed down to APOBEC3B, through the comparison of all known APOBEC mutational signatures and eliminating APOBEC3G and other deaminases from potential mutational contributors^9,13^. Soon after, APOBEC3B was found to be correlated with a variety of other cancers such as ovarian, cervical, bladder lung, head and neck; signature sequence analysis was also a contributing factor that led to these conclusions^14,15^. Most recently APOBEC3H, which has a different sequence preference than APOBEC3B, has been identified to also play a role in breast and lung cancer^16^. Thus, defining A3 sequence specificity can be helpful in identifying A3s’ role in viral restriction and in cancer.

A3 signature sequences proposed for deaminating deoxycytidines range between di-nucleotide to quad-nucleotide motifs^8–11,16–21^. A recent high-throughput assay suggested the preferred quad-nucleotide motif for A3A to be CTCG^20^. Although A3s are known to have varied sequence preference, quantitative and systematic studies of sequence specificity are incomplete. Recently, crystal structures of APOBEC3A (A3A) and APOBEC3B-CTD (an active site A3A chimera) with ssDNA have been solved^20,22^. However, despite these breakthrough structures, the molecular mechanism underlying substrate sequence specificity flanking the TC dinucleotide sequence remains unclear.

A3A is a single-domain enzyme with the highest catalytic activity among human APOBEC3 proteins^23^ and a known restriction factor for the retroelement LINE-1 and HPV^24,25^. A3A can also contribute to carcinogenesis with increased expression or defective regulation^26^. A3A is the only A3 where both the intact apo and substrate-bound structures have been determined^19,20,22,27,28^. Initial substrate specificity studies have shown selectivity for DNA over RNA, suggested by NMR chemical shift perturbation^19^. Since A3A is the best biochemically characterized A3 human cytidine deaminase and thus a critical benchmark within the family, we chose A3A to elucidate the extended characteristics of ssDNA specificity.

To determine the substrate specificity of A3A, we systematically quantified the affinity of A3A for nucleic acid substrates as a function of substrate sequence, length, secondary structure, and solution pH. We identified the A3A preferred ssDNA binding motif, (T/C)TC(A/G) and found binding correlated with enzymatic activity. Also, we determined that A3A can bind RNA in a sequence specific manner. Surprisingly, A3A’s signature sequence was necessary but not sufficient to account for A3A’s high affinity for ssDNA. Significantly, A3A bound more tightly to the motif in longer oligonucleotides, and in the context of a hairpin loop. Using recently published structures of A3As complexed with ssDNA from our lab and others, we propose a structural model for the molecular mechanism for this enhanced affinity where inter-DNA interactions contribute to A3A recognition of the cognate sequence. This model provides insights into how the nucleotides flanking the canonical TC sequence may contribute to substrate sequence preference of A3A.

## Results

### A3A binding to ssDNA is context dependent

**Figure 1:**
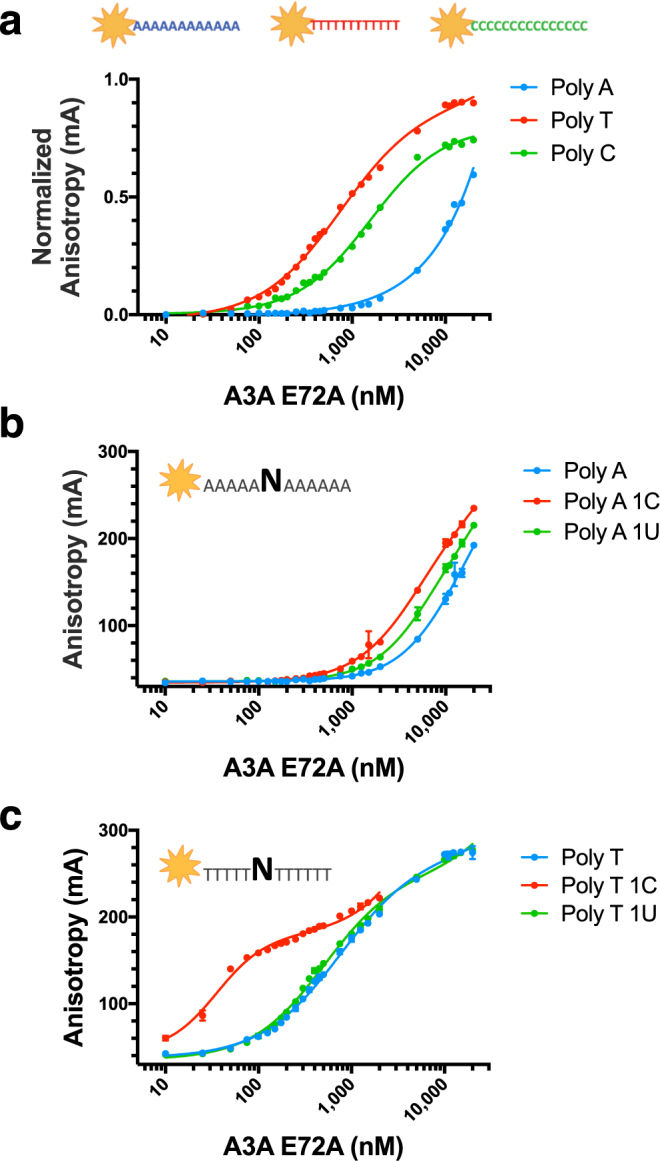
A3A specificity to ssDNA background and substrate. Fluorescence anisotropy of TAMRA-labeled ssDNA sequences binding to A3A(E72A). (**a**) Binding of A3A to poly nucleotide (12 mers): Poly A (blue), Poly T (red) and Poly C (green), (**b**) Binding to Poly A (blue), 5A-C-6A (red), 5A-U-6A (green), (**c)** Binding to Poly T (blue), 5T-C-6T (red), 5T-U-6T (green).

To interrogate the substrate sequence preference of A3A, we systematically quantified the changes in binding affinity of catalytically inactive A3A bearing the mutation E72A to a library of labeled ssDNA sequences using a fluorescence anisotropy-based DNA binding assay^28^. First, to ensure that the affinity for substrate was due entirely to the sequence of interest and not due to nonspecific binding or undesired secondary structure effects, an appropriate control background sequence was identified. The dissociation constants (K_d_’s) for homo-12-mer ssDNA sequences, Poly A, Poly T, Poly C, were determined (Fig. 1a**)**. Poly G was not tested due its propensity to form secondary structure elements. Poly T (750 ± 44 nM), which had previously been used in background sequences^28^, bound to A3A with 2-fold higher affinity than Poly C (1,600 ± 117 nM). Thus without a greater context for A3A to target, Poly C was only weakly bound. A3A had the lowest affinity for Poly A with a K_d_ of >11,000 nM (Table 1). For all subsequent assays, Poly A was used as the background, as there is no detectable binding affinity of A3A to Poly A.

**Table 1:** A3A affinity for DNA sequences used in this analysis.

DNA sequence	K_d_ (nM)
Poly C (12 C)	1,568 ± 117
Poly T (12 T)	748 ± 44
Poly T-C (5T-C-6T)	35 ± 2
Poly T-U (5T-U-6T)	499 ± 23
Poly A (12 A)	>11,000
Poly A-C (5A-C-6A)	>5,000
Poly A-U (5A-U-6A)	>6,500
Poly A-TC (4A-TC-6A)	143 ± 4
Poly A-CC (4A-CC-6A)	250 ± 14
Poly A-GC (4A-GC-6A)	>6,500
Poly A-ATUA (3A-ATUA-5A)	>5,000
Poly A-ATUG (3A-ATUG-5A)	328 ± 42
Poly A-TTUA (3A-TTUA-5A)	306 ± 17
Poly A-ATCA (3A-ATCA-5A)	145 ± 2
Poly A-ATCT (3A-ATCT-5A)	209 ± 5
Poly A-ATCC (3A-ATCC-5A)	163 ± 3
Poly A-ATCG (3A-ATCG-5A)	154 ± 2
Poly A-TTCA (3A-TTCA-5A)	90 ± 1
Poly A-TTCT (3A-TTCT-5A)	127 ± 2
Poly A-TTCC (3A-TTCC-5A)	114 ± 2
Poly A-TTCG (3A-TTCG-5A)	92 ± 2
Poly A-CTCA (3A-CTCA-5A)	85 ± 1
Poly A-CTCT (3A-CTCT-5A)	122 ± 2
Poly A-CTCC (3A-CTCC-5A)	101 ± 2
Poly A-CTCG (3A-CTCG-5A)	86 ± 1
Poly A-GTCA (3A-GTCA-5A)	150 ± 3
Poly A-GTCT (3A-GTCT-5A)	218 ± 7
Poly A-GTCC (3A-GTCC-5A)	152 ± 2
Poly A-GTCG (3A-GTCG-5A)	150 ± 3
Hairpin-TTC (G-CCATC-ATTC-GATGG-G)	26 ± 2
Hairpin-AAA (G-CCATC-AAAA-GATGG-G)	676 ± 399

The specificity of A3A for substrate versus product was measured by binding to Poly A with a single C versus Poly A with a single U (Fig. 1b). Surprisingly, the presence of a single deoxycytidine in a Poly A background was not sufficient for binding with appreciable affinity. The energetics of free ssDNA conformations in solution for Poly A sequences and base stacking propensity^29^ might be unaltered upon the introduction of a single C. The affinity of A3A for the Poly A-C (5A-1C-6A) (>5,000 nM) is similar to the affinity for Poly A-U (5A-1U-6A) (>6,500 nM) and even the background Poly A. This is in contrast to A3A’s specificity for binding a single C over U in a Poly T background, which is more than ten-fold (35 ± 2 nM and 500 ± 23 nM respectively) (Fig. 1c), as we previously measured^28^. This strong context dependence differentiating substrate C versus product U within the background of Poly A versus Poly T indicates that A3A heavily relies on the identity of the surrounding nucleotide sequence to recognize and bind substrate deoxycytidine.

### A3A affinity for ssDNA is pH dependent

A systematic measurement of A3A affinity in a broad range of pH values was performed to verify and quantify the pH dependence of A3A binding to substrate ssDNA^21,26,30^ and set a reference pH for subsequent experiments. The K_d_ of A3A for TTC in a Poly A background was determined at pH ranging from 4.0 to 9.0 in 0.5 pH increments (Supplementary Fig. 1 and Supplementary Table 1). A3A had the highest affinity for Poly A-TTC at pH 5.5 with a K_d_ of 68 ± 3 nM. The isotherms for A3A binding ssDNA at pHs below 6.0 show some secondary binding event that may be due to non-specific binding or aggregation (Supplementary Fig. 1a). A steady decrease was also observed for the affinity of A3A for ssDNA when pH was increased above 6 (Supplementary Fig. 1b), in agreement with decreased deamination activity at higher pH^26^. A3A affinity also overall correlated with reported deamination activity determined using a different assay at pH 7.5^31^. Interestingly, A3A had no appreciable affinity for Poly A-TTC above pH 8.0. Since A3A is stable at these higher pH values, the lower affinity for ssDNA with increased pH is likely not due to aggregation but due to the protonation of His29, as previously described^26^ and reported to be responsible for coordinating ssDNA^32^. Therefore, all of the subsequent binding experiments were performed at pH 6.0 to avoid any potential for secondary binding events or aggregation of the protein.

### Substrate recognition is dependent on thymidine directly upstream of target deoxycytidine, with preference for pyrimidines over purines

**Figure 2:**
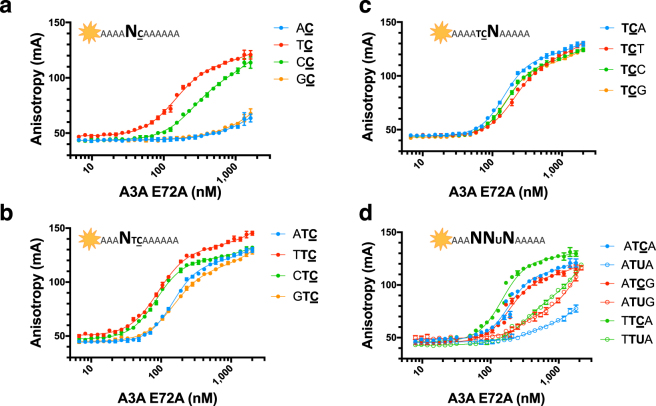
A3A specificity for nucleotides flanking substrate cytidine. Fluorescence anisotropy of TAMRA-labeled ssDNA sequences to A3A(E72A). (**a**) Binding of A3A to ssDNA with changes at −1 position of substrate C in a poly A background (12 mers): 4A-AC-6A (blue), 4A-TC-6A (red), 4A-CC-6A (green), and 4A-GC-6A (orange). (**b**) Binding of A3A to ssDNA with changes at −2 position in a TC context in a Poly A background (12 mers): 4A-ATC-6A (blue), 4A-TTC-6A (red), 4A-CTC-6A (green), and 4A-GTC-6A (orange). (**c**) Binding of A3A to ssDNA with changes at +1 position in a TC context in a Poly A background (12 mers): 4A-TCA-6A (blue), 4A-TCT-6A (red), 4A-TCC-6A (green), and 4A-TCG-6A (orange). (**d)** Three substrate sequences, TTCA (green), ATCG (red) and ATCA (blue), in closed circles with the corresponding 3 product sequences TTUA (green), ATUG (red) and ATUA (blue) in open circles.

To study the effect of the nucleotide identity at position −1 relative to target deoxycytidine (NC) on A3A affinity for substrate (Fig. 2a), the K_d_ values of A3A for (4 A)-TC**-**(6 A), AC, CC, GC in a Poly A background were determined. A preference for TC (143 ± 4 nM), followed by CC (250 ± 14 nM) was identified. Interestingly, AC and GC had similarly very weak binding affinities for A3A (>5,000 and >6,500 nM respectively), validating a preference for pyrimidines (T or C) over purines (A or G) at −1 position with T as the strongest binder.

The effects of the sequence identity around the cognate dinucleotide deamination motif (TC) on affinity of A3A for ssDNA was determined by first testing the change in affinity for all nucleotide substitutions at −2 position (3A)-NTC-(6 A). A3A has a preference for pyrimidine over purine at −2 position (Fig. 2b) with TTC and CTC having similar affinities (90 ± 1 nM and 85 ± 1 nM respectively) compared to that of purines ATC and GTC (145 ± 2 nM and 150 ± 3 nM respectively). While not as strong as for −1 position, there is a preference for the smaller pyrimidines at position −2. Next, the effect of +1 position on affinity of A3A to TC was determined (Fig. 2c). A3A did not demonstrate a strong preference for any particular nucleotide, although disfavoring T, at the +1 position (145 ± 2 nM for background versus 209 ± 5 nM).

**Figure 3:**
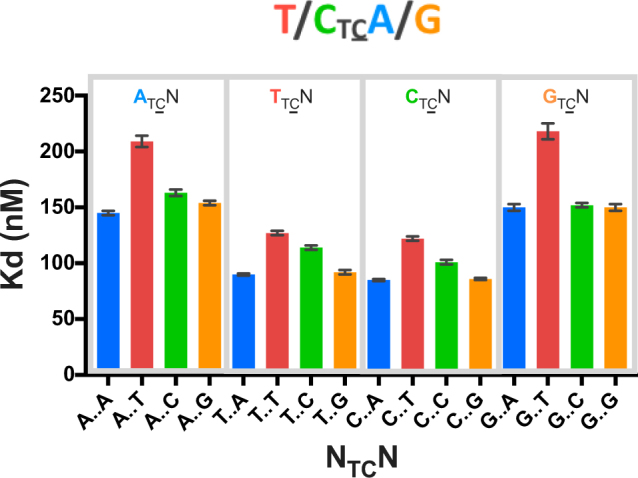
A3A specificity for poly A NTCN. Binding affinity of A3A(E72A) to TAMRA-labeled ssDNA sequences in a Poly A background. Gray boxes bin sequences by −2 nucleotide identity. Colors represent +1 nucleotide identity: A (blue), T (red), C (green), G (orange). Consensus sequence derived from these K_d_ values is shown above the graph.

Finally, to identify if there was any interdependency between nucleotide identity at −2 and +1 positions, the affinity of A3A for (3A)-NTCN-(5 A) was determined (Fig. 3, Table 1). A3A displayed preference for pyrimidines at −2 position regardless of the nucleotide at +1. A3A also disfavored T at +1 position regardless of the nucleotide identity at −2. Most interestingly, A3A preferred a pyrimidine at −2 when there was a purine at +1 position. However, the reverse was not true; purine at −2 position with pyrimidine at +1 position did not result in comparable affinities. In fact, the worst binders (ATCT and GTCT) were those that contained purines at −2 with pyrimidines at +1 position. Thus, the substrates can be broadly classified as high (80–130 nM), medium (150–165 nM), and weak (210–220 nM) affinity binders, with (T/C)TC(A/G) identified as the preferred sequence for ssDNA recognition by A3A.

A3A’s affinity for substrate C was compared to product U in the context of variations of the signature A3A substrate sequence (T/C)TC(A/G). The affinity of three substrate sequences, TTCA, ATCG and ATCA, were compared to the corresponding product sequences (Fig. 2d**)**. For all three sequences, a substantial loss of binding affinity was observed for the corresponding TTUA, ATUG and ATUA, with the most substantial loss with ATUA. Thus, the decrease in affinity for product over substrate was context dependent.

### Positive correlation between sequence preference of binding and enzymatic activity

**Figure 4:**
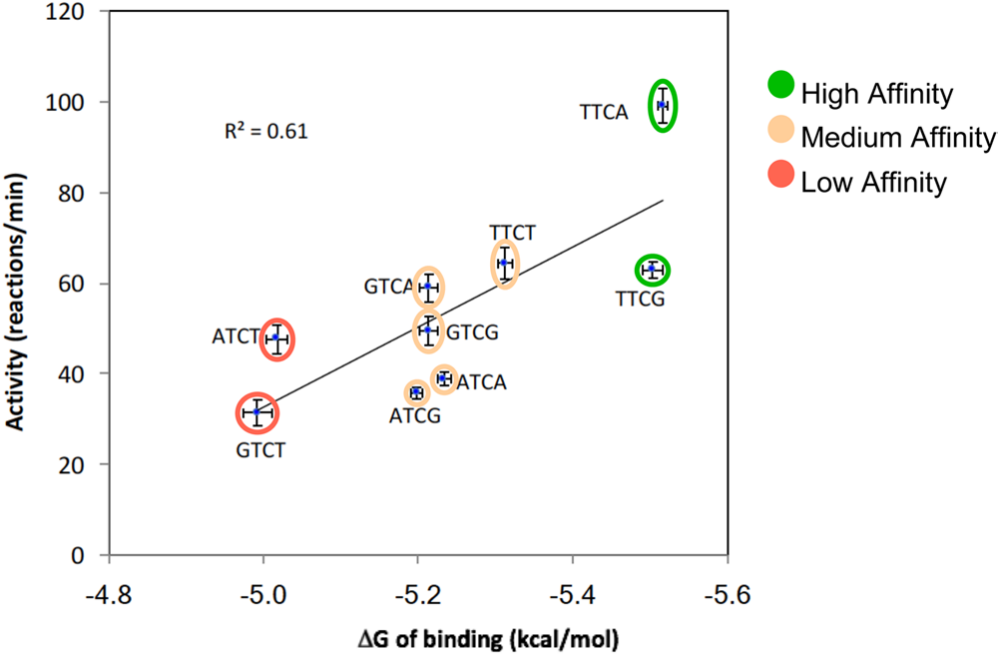
Binding affinity versus enzyme activity. The enzyme activity of active A3A measured by NMR-based deamination assay versus the free energy of binding calculated [ΔG = −RTln (K_d_)] from the binding affinity for nine 12-mers. These nine represent 2 high binding (green), 5 medium binding (orange) and 2 weak binding (red) sequences.

Although enzymatic activity and binding affinity are not expected to be directly correlated, the trends for specificity would likely be similar. The NMR assay is a highly quantitative method of observing product concentration and/or substrate reduction directly by NMR signal volumes throughout the reaction^27^. Thus A3A’s deamination activity was determined in the context of variations of the signature sequence (T/C)TC(A/G) using a ^1^H NMR based A3 deaminase activity assay. High (TTCA and TTCG), medium (ATCA, ATCG, GTCA, GTCG, TTCT) and low (ATCT and GTCT) affinity sequences were tested (Table 2) to determine the correlation between binding and activity. Overall, activity by NMR has the same trend as affinity from the binding assay (Fig. 4**)**. This indicates that in general those substrates sequences with varying binding affinity (high, medium and weak) are also processed in a similar order.

**Table 2:** A3A enzyme activity for DNA sequences.

DNA sequence	Activity (min^−1^) 40 °C
Poly A-ATCA	27 ± 1
Poly A-ATCG	28 ± 1
Poly A-ATCT	30 ± 2
Poly A-GTCA	38 ± 2
Poly A-GTCG	31 ± 2
Poly A-GTCT	22 ± 2
Poly A-TTCA	52 ± 2
Poly A-TTCG	36 ± 1
Poly A-TTCT	37 ± 2

### Structural basis for A3A specificity for binding to preferred recognition sequence

To determine the structural basis for the A3A consensus sequence (T/C)TC(A/G), crystal structures of A3A bound to ssDNA recently determined by our group and others (PDB ID: 5KEG and 5SWW) were analyzed^20,22^. The target deoxycytidine is well coordinated and buried within the active site of A3A (Supplementary Fig. 2A) in these structures. The thymidine at position −1 has extensive contacts with loop 7 (Y130, D131 and Y132), and van der Waals contacts with loop 5 (W98) (Supplementary Fig. 2B). The Watson-Crick edge of the thymidine base faces the loop 7 residues, and makes three hydrogen bonds: one with the backbone nitrogen of Y132 and the other two, one being water mediated, are with the D131 sidechain. The D131 sidechain further forms a salt bridge to the R189, which stabilizes the overall hydrogen-bonding configuration of loop 7 to the thymine base. This coordination appears critical, as residue 189 is conserved as a basic residue (Arg/Lys) in catalytically active A3 domains. This coordination also explains why −1 nucleotide must be a thymidine. If the −1 position is modeled as a cytidine, the N3 atom lacks the proton to hydrogen bond with D131 (Supplementary Fig. 2C) and would not be as well coordinated thus would be less preferable. Residues Y130 and D131 in loop 7 physically would preclude a larger purine base from fitting in this position (as modelled in Supplementary Fig. 2D). Thus the T specificity at the −1 position is consistent with the crystal structures.

**Figure 5:**
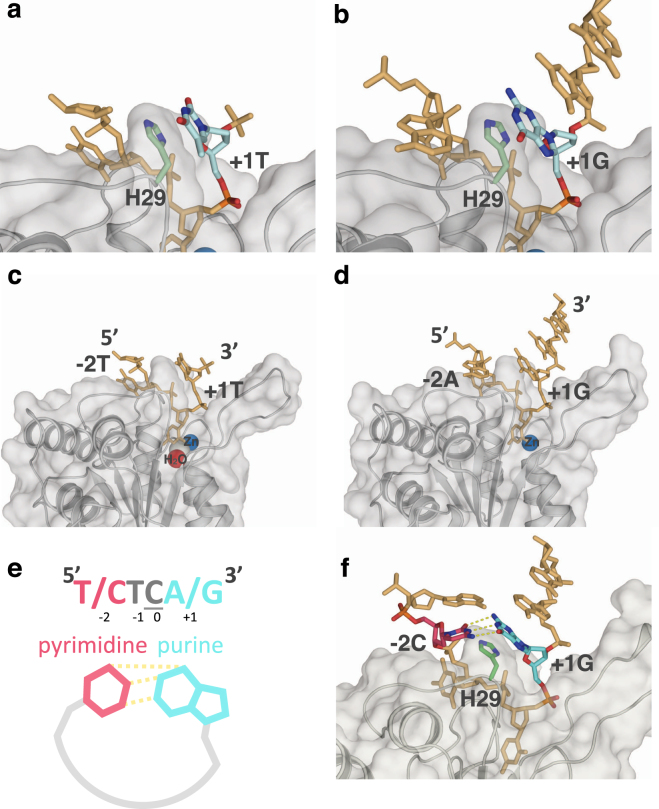
ssDNA is bent within the complex with A3A. Crystal structure of A3A shown in surface and cartoon representation (gray) bound to ssDNA displayed as orange sticks; (**a**) +1 thymidine (light blue) is interacting with His29 (light green sticks) through aromatic stacking (PDB ID: 5KEG) (**b)** +1 guanine (light blue) also interacting with His29 through aromatic stacking (light green sticks) (PDB ID: 5SWW). (**c)** A3A(E72A/C171A) with TTTTTTTTCTTTTTT (PDB ID: 5KEG) (**d**) A3A(E72A) with AAAAAAATCGGGAAA (PDB ID: 5SWW). Other nucleotides are shown as orange sticks, while water (red), zinc (blue), and chloride (gray) in the active site are shown as spheres. Nitrogen and oxygen of residues and nucleic acids are in blue and red respectively. (**e)** A schematic of hydrogen bonding between pyrimidine (pink) at −2 and purine (light blue) at +1 position via bending of the DNA by A3A upon binding. (**f)** Model of inter-DNA base interactions through binding of A3A to ssDNA. A3A(E72A)–ssDNA complex (PDB ID: 5SWW) was used to model A3A signature sequence CTCG bound at the active site. A3A is shown as gray surface and cartoon, His29 as light green sticks, original ssDNA as orange sticks with +1 G in light blue. Adenosine at −1 position was switched to cytosine (pink) with hydrogen bonds to +1 G displayed as yellow dashes.

Although A3A prefers (T/C)TC(A/G), neither of the co-crystal structures has the optimal nucleotide identity at the −2 and +1 positions^20,22^. Specificity for purine at the −2 position was not evident in the available A3A–ssDNA structures, presumably as neither structure contains an optimal ssDNA sequence. For instance, even though the 5KEG structure contains a preferred pyrimidine in the −2 position, the thymidine is disordered in this complex. However, in both structures^20,22^, the base at +1 position (pyrimidine T in 5KEG and a purine G in 5SWW) stacks with the critical His29 (Fig. 5a,b)^20,22^. This type of histidine π-π stacking can occur with either a purine or a pyrimidine. However, protonated histidine prefers to stack with a purine base over pyrimidine, with thymidine stacking being the least preferred^33^ at pH 6. Thus the base stacking potential with protonated His29 provides strong rationale for the specificity for purines and the disfavoring of thymidine at the +1 position relative to substrate deoxycytidine observed in our biochemical assays (Fig. 3).

### A3A bends ssDNA to potentially allow for intra-DNA interaction between −2 and +1 nucleotides

A common feature between the two A3A–ssDNA complex structures is that the ssDNA forms a “U” shape in the active site (Fig. 5c,d)^20,22^. This U shape of the bound polynucleotide may be conserved among deaminases, including adenosine deaminases^20,34^. In both A3A–ssDNA structures, the U shape of the ssDNA orients the −2 and +1 bases in close proximity to each other. Thus, we hypothesized that the observed sequence preference (Fig. 3) for the −2 position is a result of intra-DNA interactions rather than specific interactions with the protein.

To determine the potential for intra-DNA interactions when A3A is bound to a (T/C)TC(A/G) signature sequence, molecular models were developed based on the crystal structures of A3A bound to ssDNA (PDB ID: 5KEG and 5SWW)^20,22^. These models orient the bases of the −2 and +1 nucleotides so that they form hydrogen bonds at an angle of approximately 120 degrees and distance of less than 3.5 Å, with the larger purine at +1 position stacking on His29 and the smaller −2 pyrimidine coordinating the +1 base (Fig. 5e,f). The reversal of the nucleotides at +1 and −2 positions would not result in a fit nearly as well, which could explain the lower affinity of purine-TC-pyrimidine. Thus the structural model explains the preference for (T/C)TC(A/G) and suggests stabilizing inter-DNA interactions may further increase the affinity.

### Length of ssDNA affects affinity of A3A for substrate sequence

If the bending of the ssDNA is important for substrate recognition, dependence of binding affinity on substrate length may be expected. To determine if the DNA beyond the four-nucleotide signature sequence contributed to the binding, the length of the ssDNA that contained the recognition sequence was varied in Poly A-TTC. A competition assay with different length oligonucleotides was performed to test the effect of ssDNA length on affinity for substrate (Supplementary Fig. 3). Length was varied from 1 nucleotide flanking each end of TTCA (TTCAA and ATTCA) to 3 nucleotides flanking each end, increasing by one nucleotide addition on either end. Surprisingly, a single nucleotide flanking TTCA signature sequence was not enough to permit binding (Supplementary Fig. 3a), and even three nucleotides on either side still did not bring A3A binding to original binding affinity as Poly A-TTC (AAA TTCA AAA AAA) (Supplementary Fig. 3b). Thus, binding affinity is impacted beyond the recognition motif to prefer longer sequences, although the additional nucleotides are not expected to have any direct contacts with A3A, consistent with the model that intra-DNA interactions modulate A3A affinity.

### A3A prefers binding to target sequence in the loop of structured hairpins

**Figure 6:**
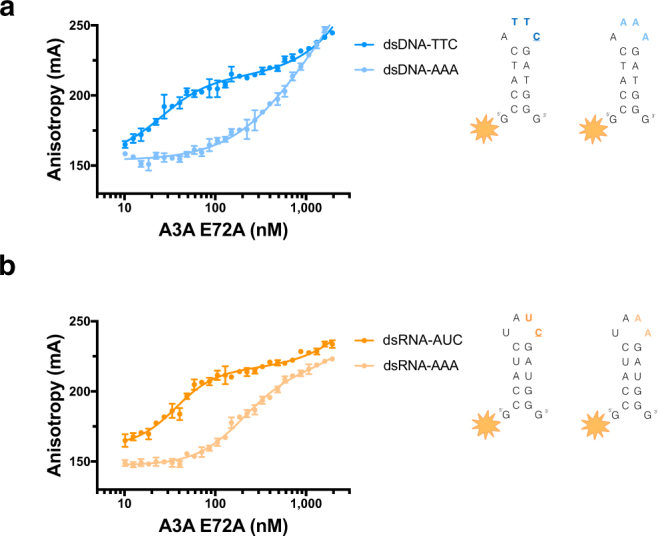
A3A specificity for substrate in loop region of stem-loop (hairpin) nucleic acids. Fluorescence anisotropy of TAMRA-labeled hairpin DNA and RNA binding to A3A(E72A). (**a**) Binding of A3A to a DNA version of the hairpin SDHB RNA containing TTC (dark blue) and AAA (light blue) in the loop region. (**b**) Binding of A3A to hairpin SDHB RNA (dark orange) and the same RNA sequence replacing the UC with AA in the loop region of the hairpin (light orange).

Another implication of this model would be that pre-bent DNA could be a better substrate for A3A binding, as A3A would not have to pay the entropic cost of bending the DNA. This bending of DNA could be achieved either by the inter-DNA interactions modeled in Fig. 5f, or when within a loop of a hairpin. To determine the significance of the bent DNA structure in the mechanism of A3 binding, we tested A3A affinity to a target deoxycytidine in the loop region of a DNA hairpin. The hairpin sequence was based on a previously identified potential RNA substrate for A3A, from succinate dehydrogenase complex iron sulfur subunit B (SDHB)^35^. The affinity for TTC in the loop region of this hairpin DNA was higher than that in linear DNA (26 nM vs 90–127 nM respectively). As expected, A3A had a higher affinity for the DNA hairpin with loop region containing TTC compared to one with AAA (26 nM vs ~676 nM respectively) (Fig. 6a). Interestingly, the K_d_ value for the hairpin (26 nM) is comparable to that for a single C in a polyT background (35 nM)^28^. This may imply that the Poly T DNA adopts a hairpin structure in solution, as has been reported^36^.

A3A affinity to a target cytidine in the loop region of an RNA hairpin was also tested. The exact SDHB hairpin RNA sequence including UC in the loop of this hairpin versus a modified SDHB hairpin RNA replacing the AUC with AAA was compared. A3A had specific affinity for the hairpin RNA containing UC compared to AA (37 nM vs 202 nM respectively) (Fig. 6b). In contrast to what has been previously proposed^19^, we found that A3A has high affinity and specificity for RNA. Furthermore, A3A has a higher affinity for AUC in the loop region of a hairpin compared to UUC in a linear sequence (Supplementary Fig. 4). The potential UUC substrate sequence in linear RNA has no measurable affinity, comparable to linear RNA without a potential substrate sequence. Overall, A3A has higher affinity for target sequence in the context of a pre-ordered loop region rather than linear DNA, and specific affinity for RNA hairpins with a substrate site.

## Discussion

A3A is a single-domain enzyme with the highest catalytic activity among the human APOBEC3 proteins^23^, a known restriction factor^24,25^, and also likely contributes to carcinogenesis^26^. In this study we quantified the ssDNA specificity of A3A, and identified the consensus signature sequence as (T/C)TC(A/G). The dinucleotide sequence preference for A3A, TC, which was previously found through activity assays^10,20,21^ was confirmed and expanded to a preference for pyrimidine-TC-purine. Surprisingly context matters, in that the background nucleotide sequence impacts binding affinity, with essentially no binding observed for Poly A 1 C **(**Fig. 1b), while Poly T 1 C binds with 35 ± 2 nM affinity^28^. Furthermore, the length of the ssDNA in which (T/C)TC(A/G) is imbedded within also modulates affinity (Supplementary Fig. 3). Structural analysis of the two A3A–ssDNA complexes containing two distinct, but suboptimal ssDNA sequences have led us to develop a model with intra-DNA interactions for the molecular mechanism for A3A’s specificity to ssDNA. In contrast to previous results^27^, which implicate the −2 position as defining specificity, the base at this position observed in both A3A–ssDNA co-crystal structures do not make any specific interactions with the protein. Rather, the hydrogen bonding edge of the −2 base is in close proximity to corresponding edge of +1 base, suggesting possible intra-DNA interactions as being determinants of preference. Our molecular modeling confirmed such interactions could stabilize the U-shaped DNA conformation within the A3A active site, explaining the −2 position specificity.

We found that A3A binds to RNA in a highly specific and structural context-dependent manner. Previous reports^19^ suggested that A3A bound only weakly and did not deaminate RNA. However, the potential substrate sequence was designed to lack secondary structure, which in light of our results on hairpin versus linear RNAs, may have inadvertently precluded RNA deamination. Recently, A3G and A3A were implicated in deaminating RNA in proposed RNA hairpins in whole cell lysates but the specificity was not quantified^35,37^. Intriguingly, our data show that A3A binds RNA hairpins with similar affinity as for DNA hairpins, which suggests that RNA-editing activity of A3A might be more prevalent than previously anticipated. Future experiments will identify if A3A’s catalytic efficiency is similar for DNA and RNA hairpins.

The comprehensive identification of A3A signature sequences and preference for loop structures will enable a more accurate evaluation of A3 activity based on sequence analysis. Previous studies used only a *single* identified A3 signature sequence to implicate A3’s role in viral restriction or cancer progression. In contrast, our study suggests a more accurate method for determining evidence of A3 activity would be to use a set of sequences. In the case of A3A, we have identified four almost equivalent substrate signature sequences, TTCA, TTCG, CTCA, and CTCG, which should be used for identifying A3A’s involvement in mutagenesis. We also found a positive correlation between A3A’s sequence preference of binding and enzymatic activity. This correlation not only legitimizes the use of a DNA binding assay with inactive enzyme as a reliable method for studying specificity of A3s, it also shows that affinity for substrate is a driving factor for catalysis. Thus, factors that could enhance or perturb binding, such as pH or nucleic acid structure, would result in modulation of deamination activity.

In addition to using the full A3A signature sequences, the probability of mutagenesis should not be solely based on nucleotide sequence, but should also be weighted by the propensity of the target sequence to be within a structured loop. Secondary structure prediction software could be used to identify the consensus sequence in loop regions of structured DNA or RNA. A3A signature sequences that we identified, (T/C)TC(A/G), not only account for the discrepancies in the A3A target sequences reported in the literature such as TTCA versus CTCG^20,21^, but also lead us to advocate a new paradigm for identifying A3A’s involvement in mutation of endogenous or exogenous DNA.

Designing inhibitors or activators for A3s has been extremely challenging. Our results implicate a need to incorporate the structural context of the target deoxycytidine in the therapeutic design. Larger macrocycles may serve as more appropriate starting scaffolds in designing cancer therapies targeting A3s, which would mimic the “U” shape of the bound ssDNA. Macrocycles have recently been shown to have good drug-like properties and may be a strategy to target these critical enzymes^38^.

## Material and Methods

### Cloning of APOBEC3A E72A overexpression construct

The pColdII His-6-SUMO-A3A(E72A) was constructed by first cloning the SUMO gene from pOPINS His-6-SUMO into pColdII His-6 vector (Takara Biosciences) using NdeI and KpnI restriction sites. Human APOBEC3A coding sequence from pColdIII GST-A3A(E72A, C171A) was then cloned into the pColdII His-6-SUMO vector with KpnI and HindIII. The C171A mutation in the A3A construct was reverted to wild type residue by site directed mutagenesis resulting in the pColdII His-6-SUMO-APOBEC3A(E72A) catalytically inactive over-expression construct used in this study.

### Expression and purification of APOBEC3A E72A

Escherichia coli BL21 DE3 Star (Stratagene) cells were transformed with the pColdII His-6-SUMO-APOBEC3A(E72A) vector described above. The E72A mutation was chosen to render the protein inactive. Expression occurred at 16 °C for 22 hours in lysogeny broth medium containing 0.5 mM IPTG and 100 µg/mL ampicillin. Cells were pelleted, re-suspended in purification buffer (50 mM Tris-HCl [pH 7.4], 300 mM NaCl, 1 mM DTT) and lysed with a cell disruptor. Cellular debris was separated by centrifugation (45,000 g, 30 min, 4 °C). The fusion protein was separated using HisPur Ni-NTA resin (Thermo Scientific). The His-6-SUMO tag was removed by means of a Ulp1 protease digest overnight at 4 °C. Untagged A3A(E72A) was separated from tag and Ulp1 protease using HisPur Ni-NTA resin. Size-exclusion chromatography using a HiLoad 16/60 Superdex 75 column (GE Healthcare) was used as a final purification step. Purified recombinant A3A was determined to be free of nucleic acid prior to binding experiments by checking OD 260/280 ratios, which was at 0.54.

### Oligo source and preparation

Labeled and unlabeled oligonucleotides used in this assay were obtained through Integrated DNA Technologies (IDT). Labeled oligonucleotides used in the fluorescence anisotropy based binding assay contain a 50-TAMRA flourophore at their 5′ end and were resuspended in ultra-pure water at a concentration of 20 µM. Unlabeled oligonucleotides used for the competition assays were resuspended in ultra-pure water to a concentration of 4 mM.

### Fluorescence anisotropy based DNA binding assay

Fluorescence anisotropy based DNA binding assay was performed as described^28^ with minor alterations. A fixed concentration of 10 nM 50-TAMRA-labeled oligonucleotides was added to A3A(E72A) in 50 mM MES buffer (pH 6.0), 100 mM NaCl, 0.5 mM TCEP in a total reaction volume of 150 µL per well in nonbinding 96-well plates (Greiner). The concentration of A3A was varied in triplicate wells. Plates were incubated for overnight at room temperature.

For the pH dependence experiments the buffer reagent used for testing was pH 4.0–5.0 sodium acetate, pH 5.5–6.5 MES, pH 7.0–8.0 HEPES, pH 8.5–9.0 TRIS. Assay was performed as described above. For the competition assays, a fixed concentration of 300 nM A3A(E72A) was used and unlabeled oligonucleotide of varied concentration was added from 0–6.1 μM. A3A(E72A) was pre-incubated with unlabeled oligonucleotide for an hour in assay buffer, then labeled DNA was added and incubated overnight at room temperature.

For all experiments, fluorescence anisotropy was measured using an EnVision plate reader (PerkinElmer), exciting at 531 nm and detecting polarized emission at 579 nm wavelength. For analyzing data and determining K_d_ values, Prism (GraphPad) was used for least-square fitting of the measured fluorescence anisotropy values (Y) at different protein concentrations (X) with a single-site binding curve with Hill slope, a nonspecific linear term, and a constant background using the equation ${\rm{Y}}=(({{\rm{Bmax}}}^{\ast }{{\rm{X}}}^{\wedge }{\rm{h}})/({{\rm{Kd}}}^{\wedge }{\rm{h}}+{{\rm{X}}}^{\wedge }{\rm{h}}))+{{\rm{NS}}}^{\ast }{\rm{X}}+{\rm{Background}}$, where K_d_ is the equilibrium dissociation constant, h is the Hill coefficient, and Bmax is the extrapolated maximum anisotropy at complete binding.

### ^1^H NMR based A3 deaminase activity assay

Deaminase activity was determined for A3A protein by assaying active enzyme against linear DNA substrates and measuring the product formation using ^1^H NMR. Active A3A protein (50 nM) was assayed against linear DNA substrates (200 µM) in buffer with 50 mM MES pH 6.0, 100 mM NaCl, 0.5 mM TCEP, and 5% D_2_O. Experiments were performed on 9-mer substrates containing the target sequences AA(A/G/T)TC(A/G/T)AAA and at 40 °C to prevent the DNA from oligomerizing due to high concentration. Experiments were performed using a Bruker Avance III NMR spectrometer operating at a ^1^H Larmor frequency of 600 MHz and equipped with a cryogenic probe. Product concentration was estimated from peak integrals with Topspin 3.5 software (Bruker Biospin Corporation, Billerica, MA) using an external standard. Activity was determined from the initial rate of product formation via first-order exponential fitting of the progress curve. Rate errors were estimated by Monte Carlo simulation using 100 synthetic data sets and taking the residuals of the initial fit to the experimental data as the concentration error.

### Molecular Modeling

The crystal structures of A3A bound to ssDNA (PDB ID: 5KEG and 5SWW) were used for molecular modeling^20,22^. The DNA sequence was first mutated using Coot^39^. The complex structure was then prepared, energy minimized with ProteinPrep Wizard in Maestro (Schrödinger) using the OPLS3 force field, at pH 6.0 with all other settings kept as default.

## Electronic supplementary material

Supplementary Information
