# Supplementary material for: Substrate sequence selectivity of APOBEC3A implicates intra-DNA interactions
Source: Sci Rep. 2018 May 14;8:7511. doi: 10.1038/s41598-018-25881-z (PMC5951847; doi:10.1038/s41598-018-25881-z)
Supplement: Supplementary file 1 — Supplementary Information [file 41598_2018_25881_MOESM1_ESM.pdf]

Supplementary Information

Substrate sequence selectivity of APOBEC3A implicates intra-DNA interactions

Tania V. Silvas[1], Shurong Hou[1], Wazo Myint[2], Ellen Nalivaika[1], Mohan Somasundaran[1], Brian A. Kelch[1], Hiroshi Matsuo[2], Nese Kurt Yilmaz[1], and Celia A. Schiffer[1,*]

[1]Biochemistry and Molecular Pharmacology, UMass Medical School, Worcester, MA, 01655, USA
[2]Leidos Biomedical Research, Inc., Frederick National Laboratory for Cancer Research, Frederick, MD, 21701, USA

* To whom correspondence should be addressed.

Email: Celia.Schiffer@umassmed.edu

Celia Schiffer, Ph.D.
Department of Biochemistry and Molecular Pharmacology
University of Massachusetts Medical School
364 Plantation Street
Worcester MA, 01605-2324
Phone: (508) 856-8008
Fax: (508) 856-6215

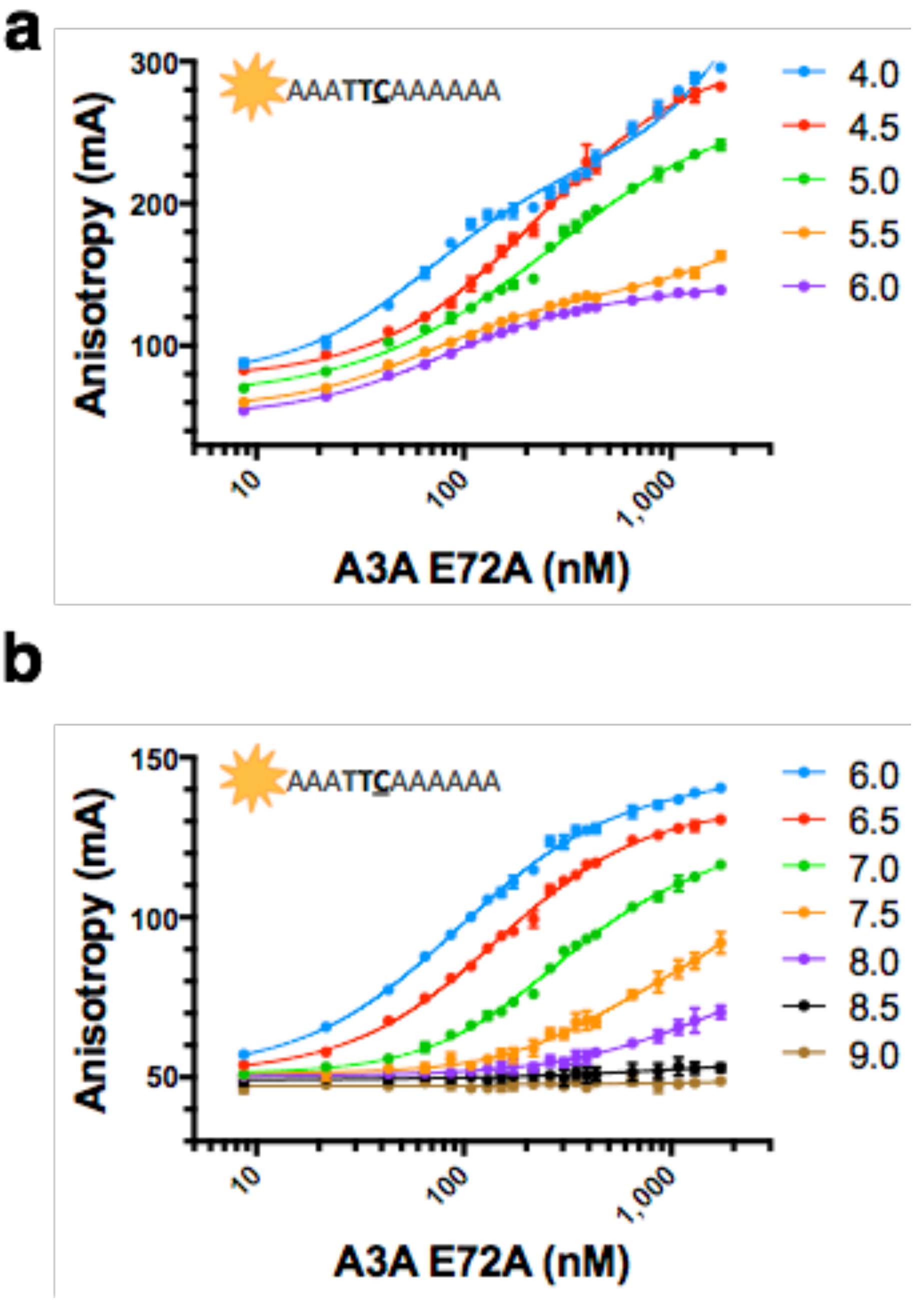

<u>Supplementary Figure 1.</u> A3A affinity to ssDNA at different pHs. Fluorescence anisotropy of TAMRA-labeled ssDNA 4A-TTC-6A binding to A3A(E72A). **a)** Binding of A3A to ssDNA at pH 4.0 (blue), 4.5 (red), 5.0 (green), 5.5 (orange), and 6.0 (purple). **b)** Binding of A3A to ssDNA at pH 6.0 (blue), 6.5 (red), 7.0 (green), 7.5 (orange), 8.0 (purple), 8.5 (black), 9.0 (brown).

Supplementary Table 1. A3A affinity (K_d in nM) for ssDNA Poly A -TTC in a range of pHs.

pH	KD
**4.0**	Could not calculate
**4.5**	Could not calculate
**5.0**	Could not calculate
**5.5**	68 ± 3
**6.0**	90 ± 1
**6.5**	146 ± 8
**7.0**	260 ± 21
**7.5**	> 4,500
**8.0**	> 5,000
**8.5**	no detectable binding
**9.0**	no detectable binding

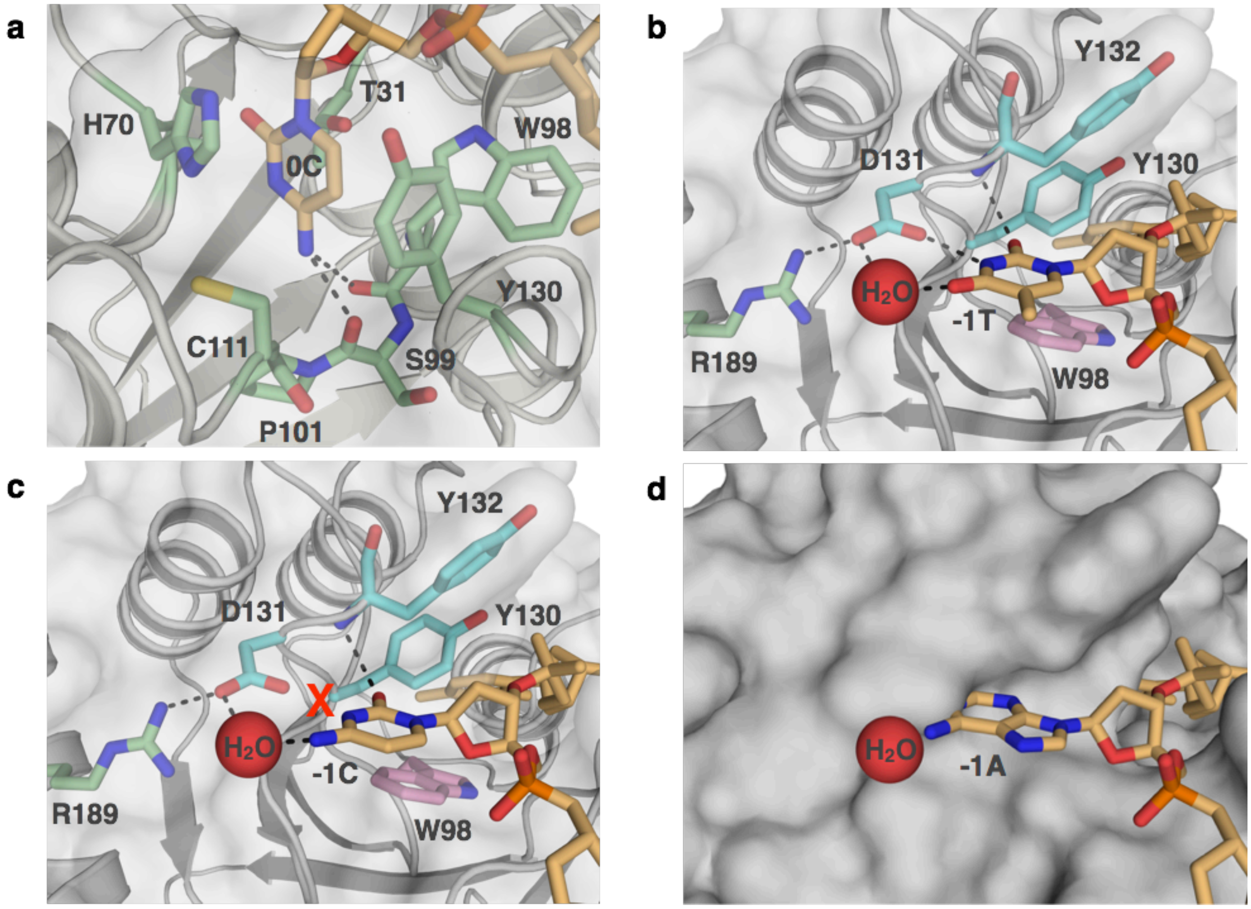

<u>Supplementary Figure 2.</u> **A3A recognition of substrate cytidine and pyrimidines at -1.** Crystal structure of A3A(E72A/C171A) shown in surface view (gray) bound to Poly T-1C ssDNA sequence represented as sticks (PDB ID: 5KEG). **a)** substrate cytidine (orange sticks) is buried in active site of A3A. Residues interacting with cytidine are shown in green sticks. **b)** -1 nucleotide thymidine (orange sticks) surrounded by Y130, D131 and Y132 of loop 7 (light blue sticks), W98 of loop 5 (pink sticks), and R189 (green sticks). **c)** Cytidine modeled into -1 position (orange sticks). N3 atom lacks proton to hydrogen bond with D131 indicate with a red X. **d)** Adenosine modeled into -1 position (orange sticks) shows severe van der Waal clashes if occupying the same site as the pyrimidines. Other nucleotides are shown as orange sticks. Hydrogen bond and a salt bridges shown in dashes black lines. Water shown as red spheres. Nitrogen and oxygen of residues and nucleic acids are in blue and red respectively.

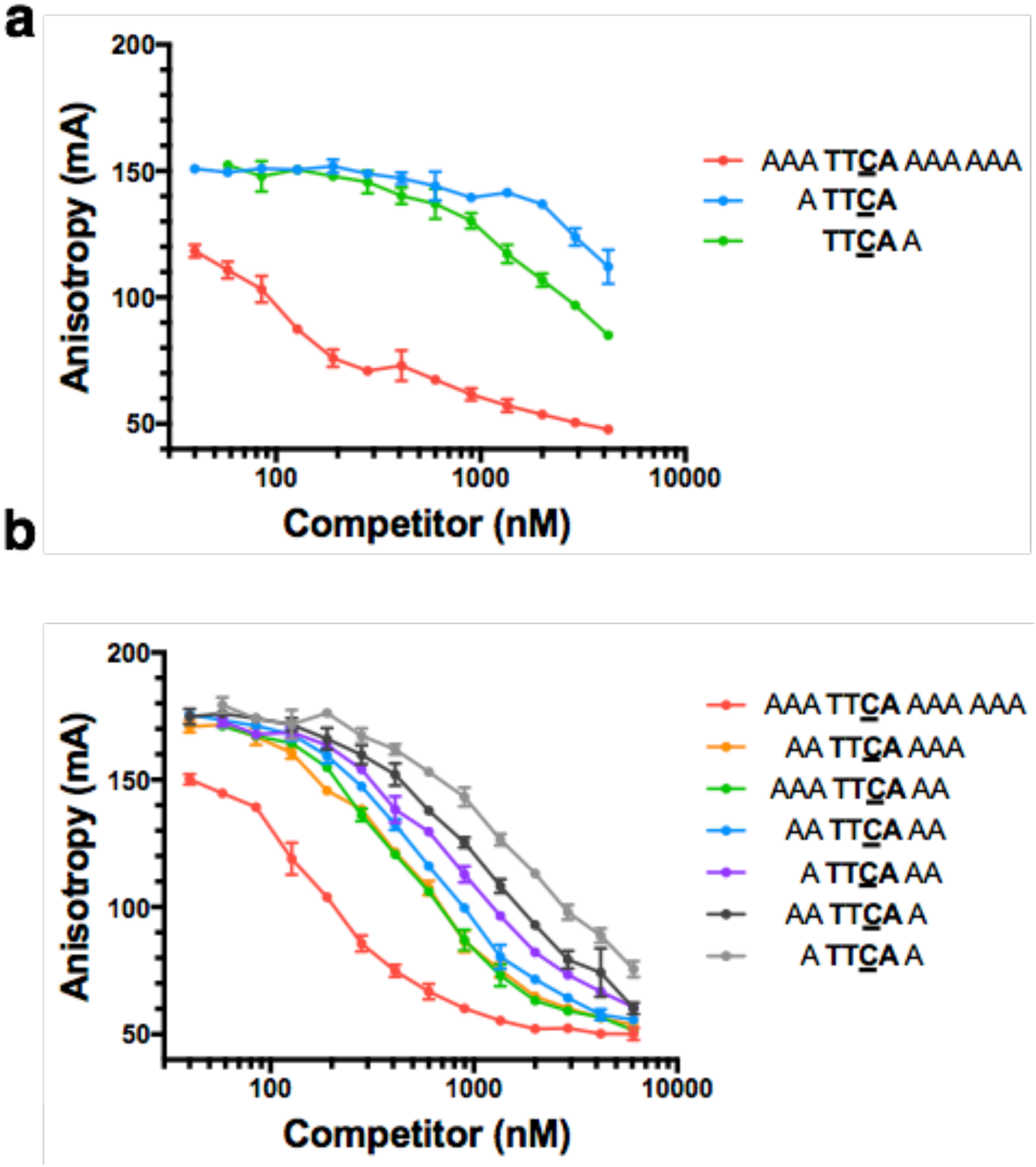

<u>Supplementary Figure 3.</u> A3A affinity to ssDNA of varied lengths. Fluorescence anisotropy of TAMRA-labeled ssDNA 3A-TTCA-6A to A3A(E72A) competing with unlabeled ssDNA of different lengths. **a)** Binding of A3A to labeled ssDNA preincubated with unlabeled 3A-TTCA-6A (red), 1A-TTCA (blue), and TTCA-1A (green). **b)** Binding of A3A to labeled ssDNA preincubated with unlabeled 3A-TTCA-6A (red), 2A-TTCA-3A (blue), 3A-TTCA-2A (green), 2A-TTCA-2A (blue), 1A-TTCA-2A (purple), 2A-TTCA-1A (black), and 1A-TTCA-1A (gray).

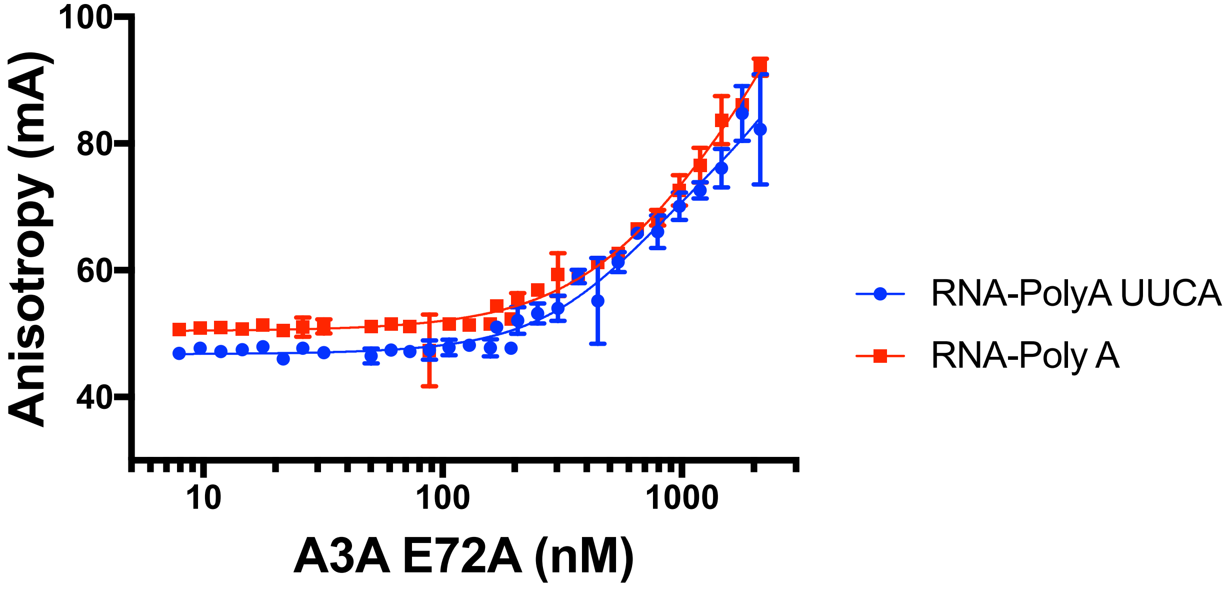

Supplementary Figure 4. A3A affinity to ssRNA
Fluorescence anisotropy of TAMRA-labeled ssRNA sequences to A3A(E72A). Binding of A3A to ssRNA with PolyA UUCA (blue) and ssRNA Poly A (red).
